# Performance of the SD BIOLINE® HAT rapid test in various diagnostic algorithms for *gambiense* human African trypanosomiasis in the Democratic Republic of the Congo

**DOI:** 10.1371/journal.pone.0180555

**Published:** 2017-07-03

**Authors:** Crispin Lumbala, Paul R. Bessell, Pascal Lutumba, Sylvain Baloji, Sylvain Biéler, Joseph M. Ndung'u

**Affiliations:** 1 PNLTHA, Bâtiment PNMLS, 1 Boulevard Triomphal, Kinshasa, Democratic Republic of the Congo; 2 Epi Interventions Ltd, 32 Bell Place, Edinburgh, United Kingdom; 3 Faculty of Medicine, University of Kinshasa, Kinshasa, Democratic Republic of the Congo; 4 Institute National de Recherche Biomédicale, Kinshasa, Democratic Republic of the Congo; 5 Foundation for Innovative New Diagnostics (FIND), Campus Biotech, 9 Chemin des Mines, Geneva, Switzerland; Universidade Nova de Lisboa Instituto de Higiene e Medicina Tropical, PORTUGAL

## Abstract

We carried out a study to compare the performance, in terms of sensitivity and specificity, of the new SD BIOLINE^®^ HAT rapid diagnostic test (RDT) with the card agglutination test for trypanosomiasis (CATT) for diagnosis of human African trypanosomiasis (HAT) in the Democratic Republic of the Congo (DRC). Participants were enrolled actively by four mobile teams, and passively at four health facilities in three provinces. Consenting participants were tested concurrently with the RDT and CATT on whole blood. Those found positive by either test were tested with CATT on serial dilutions of plasma, and with a parasitological composite reference standard (CRS). Cases were only the individuals found positive by the CRS, while controls were negative by both CATT and RDT, as well as those that were positive by CATT or RDT, but were negative by the CRS, and had no history of HAT. Over five months, 131 cases and 13,527 controls were enrolled. The sensitivity of the RDT was 92.0% (95% confidence interval (CI) = 86.1–95.5), which was significantly higher than CATT (sensitivity 69.1%; 95% CI = 60.7–76.4). The sensitivity of CATT on plasma at a dilution of 1:8 was 59.0% (95% CI = 50.2–67.2). The specificity of the RDT was 97.1% (95% CIs = 96.8–97.4) while that of CATT was 98.0% (95% CIs = 97.8, 98.2) and specificities of algorithms involving CATT at 1:8 dilution were 99.6% (95% CI = 99.5–99.7). Reproducibility of results was excellent. We concluded that an algorithm in which the SD BIOLINE^®^ HAT RDT is used for screening is optimal for case detection in both passive and active screening settings. However, the lower specificity of the RDT compared to that of CATT would result in a larger number of false positive individuals undergoing confirmatory testing.

## Introduction

Human African trypanosomiasis (HAT), also known as sleeping sickness, is a vector-borne parasitic disease transmitted to humans by the bite of an infected tsetse fly (*Glossina spp*). The disease is endemic in sub-Saharan Africa, within the limits of the geographic distribution of the tsetse fly. Two sub-species of the protozoan parasite *Trypanosoma brucei* cause the disease in humans: *T*.*b*. *gambiense* and *T*.*b*. *rhodesiense*. Infection with *T*.*b*. g*ambiense* causes the chronic form of HAT (*gambiense* HAT) and accounts for more than 95% of cases [1]. *Gambiense* HAT is endemic in rural, resource-limited settings, mainly in west and central Africa, with the majority of cases reported in the Democratic Republic of the Congo (DRC) [2]. The number of cases of HAT reported globally has been falling steadily, and the disease is now targeted for elimination as a public health problem by 2020 [3].

Control of *gambiense* HAT is based on detection and treatment of infected individuals and early and accurate diagnosis of the disease is essential, as early treatment is easier, safer, and more effective [4,5], while early detection truncates the window for onward transmission. However, the early stage of *gambiense* HAT is often sub-clinical and once clinical signs appear, they are similar to those of malaria, a disease that is endemic in all regions where HAT occurs. As a consequence, it is necessary to screen a large number of people among the population at risk in order to identify HAT cases. The card agglutination test for trypanosomiasis (CATT) is at present the most commonly used test for screening HAT [6]. CATT is a serological test that detects host antibodies to infection, with a sensitivity of around 90% and specificity of between 97–99% [1,7–9]. The specificity of the test can be improved if it is repeated on serially diluted plasma from individuals who are positive on whole blood [10]. People who are positive by the screening test are submitted to confirmatory tests that are based on visualisation of trypanosomes by microscopy in lymph node aspirates, blood or cerebrospinal fluid (CSF). Due to the relatively low density of parasites in the blood of HAT cases, concentration techniques such as the micro-haematocrit centrifugation technique (mHCT or Woo test) and the mini-anion exchange centrifugation technique (mAECT) are used to enhance sensitivity by improving the likelihood that parasites will be visualised [11]. Following confirmation, the patient is treated according to the stage of disease, which is determined by performing a lumbar puncture and examining the CSF for the presence of trypanosomes, and counting the number of white blood cells (WBC) [12–14]. During early or stage 1 of the disease, parasites are found only in the haemolymphatic system, while the advanced or stage 2 disease is associated with presence of parasites in the CSF and/or more than 5 WBCs per μl [4].

While CATT has been widely used by vehicle- and boat-based mobile teams, including in the DRC [12], its use for passive screening is associated with a number of constraints. These include the requirement for a source of power and a cold-chain, and the 50 dose format of CATT means that when the reagent is reconstituted, the doses must be used within a few days in order to avoid spoilage [15,16]. To reduce this wastage, the Institute of Tropical Medicine (ITM) developed the CATT D10, which contains reagents for 10 tests [16,17]. Whilst the CATT D10 is thermostable, the vial of reagent must be used within 24 hours after opening, which has restricted its widespread use by national HAT control programmes. As an alternative to CATT, two rapid diagnostic tests (RDTs) have recently been developed and commercialized. These are the SD BIOLINE^®^ HAT RDT developed by Alere/Standard Diagnostics, Inc. (SD) in collaboration with the Foundation for Innovative New Diagnostics (FIND) [18], and the HAT Sero-K-Set developed by Coris BioConcept in collaboration with the ITM [19,20]. Both are first generation RDTs based on native antigens, and at the time of writing, were the only HAT RDTs that have been commercialised. RDTs are performed on fresh blood from a finger prick, do not require any instrument, and test results are obtained in 15 minutes. With these characteristics, RDTs could play a major role in both screening for HAT at health facilities at the lowest level of the healthcare system, and in active screening by health workers at the level of the community.

The SD BIOLINE^®^ HAT RDT is stable for at least 24 months at 40^°^C, and detects antibodies against two trypanosome variable surface glycoprotein (VSG) antigens (LiTat 1.3 and LiTat 1.5) which are incorporated as separate bands. When a test is performed and any of the test bands is observed, the result is interpreted as positive. The CATT on the other hand detects antibodies against *T*. *b*. *gambiense* parasites expressing VSG LiTat 1.3. A prototype of the SD BIOLINE^®^ HAT RDT was found to have a sensitivity of 89.3% and a specificity of 94.6%, but its performance varied by geographic settings [21]. The sensitivity was not significantly different to that of CATT on whole blood (p = 0.21) or CATT on plasma at a dilution of 1:8 (p = 0.85). The specificity of the prototype RDT was 94.6%, which was significantly lower (p<0.001) than that of CATT on whole blood (95.9%), and significantly lower (p<0.001) than the specificity of CATT on plasma at a dilution of 1:8 (98.9%) [21]. Based on these results, the SD BIOLINE^®^ HAT RDT was optimized further to improve sensitivity, without compromising specificity. This was done at the level of manufacturing, by changing the composition of buffer that is used in the test, and validated by testing stored samples from 49 HAT cases and 399 HAT negative controls.

This study was carried out to evaluate the performance (sensitivity, specificity and reproducibility) of the SD BIOLINE^®^ HAT RDT in field settings in the DRC, by comparing the RDT and CATT as the screening test in both active (by mobile teams) and passive (in health facilities) settings. This was part of a large study to demonstrate the use of the HAT RDT as part of the routine screening activities of the national HAT control programme of the DRC.

## Materials and methods

### Study sites

This study was carried out in the provinces of Bandundu (Kwamouth and Bagata general hospitals, Kwamouth and Bagata mobile teams; now in Mai-Ndombe and Kwilu provinces), East Kasaï (Lukalaba hospital and Miabi mobile team) and West Kasaï (Kakenge reference health centre and Kakenge mobile team, now Kasai province) in the DRC. Study sites were visited by an external monitor prior to commencement of the study to verify that they were adequately prepared and staff were properly trained, and during the study, they were visited to ensure that the protocol was being adhered to.

### Enrolment of participants

Participants were enrolled passively in the 4 health facilities, and actively by the 4 mobile teams. In health facilities, participants were enrolled from among patients presenting themselves or referred from other health facilities after suspicion of HAT or other diseases, and among relatives who accompanied patients. During active screening, all those who presented themselves to the mobile team were eligible for enrolment in the study. From those found positive by the RDT or CATT, written informed consent was sought. No additional information or samples were collected from those that were negative by RDT and CATT; only a count of the numbers screened for use in specificity analysis, and hence there was no requirement for informed consent. People who presented for screening but did not wish to participate in the study were screened according to the procedures of the national programme. All consented participants were tested for malaria and if found positive, they were managed according to the guidelines of the national malaria control programme, but remained eligible for enrolment in this study.

### Tests performed

The CATT and HAT RDT were performed on fresh blood obtained from a finger prick for each individual presenting for active or passive screening. The results of CATT and RDT were each read independently by two laboratory technicians or nurses who were specialised in HAT, and the results were recorded separately. The technicians were blinded to the result of one another. For the RDT, the overall result of the RDT was recorded as per the manufacturer’s guidelines. A positive result is a reaction on either of the two bands corresponding to VSG LiTat 1.3 (band 1) or VSG LiTat 1.5 (band 2), whilst a reaction on the control band alone is a negative result. The intensity of each RDT band was qualitatively assessed and scored from 0 to 4 according to a printed scale that was provided by the manufacturer, with 0 equating to absence of a band. The overall result and the result on individual bands were recorded separately. The result of CATT was recorded simply as positive or negative, but a note was made for any CATT result whose interpretation was deemed to be a doubtful result.

Participants found positive by either reader by CATT or the RDT were tested with a parasitological composite reference standard (CRS), which involved carrying out a number of parasitological tests that are in standard use in the DRC. To perform the CRS, 5 ml venous blood was collected, and patients with palpable cervical lymph nodes had a lymph node aspirate taken and examined for motile parasites by bright field microscopy. If lymph node palpation was not possible or was negative, 500 μl of blood was used to perform the mini anion exchange centrifugation technique (mAECT) and 4 capillary tubes of 75 μl each were used for the micro-haematocrit centrifugation technique (mHCT or Woo test) (Study protocol S2 File). The technician performing parasitology was blinded to the results of CATT and RDT, and any samples that were positive by a parasitological technique were verified by the unit’s supervisor.

If the subject was positive by CATT, the remaining blood was centrifuged and 30 μl of plasma used to prepare dilutions for repeat testing with CATT. The results of CATT dilutions were also read by two technicians independently, and recorded separately.

**Fig 1:**
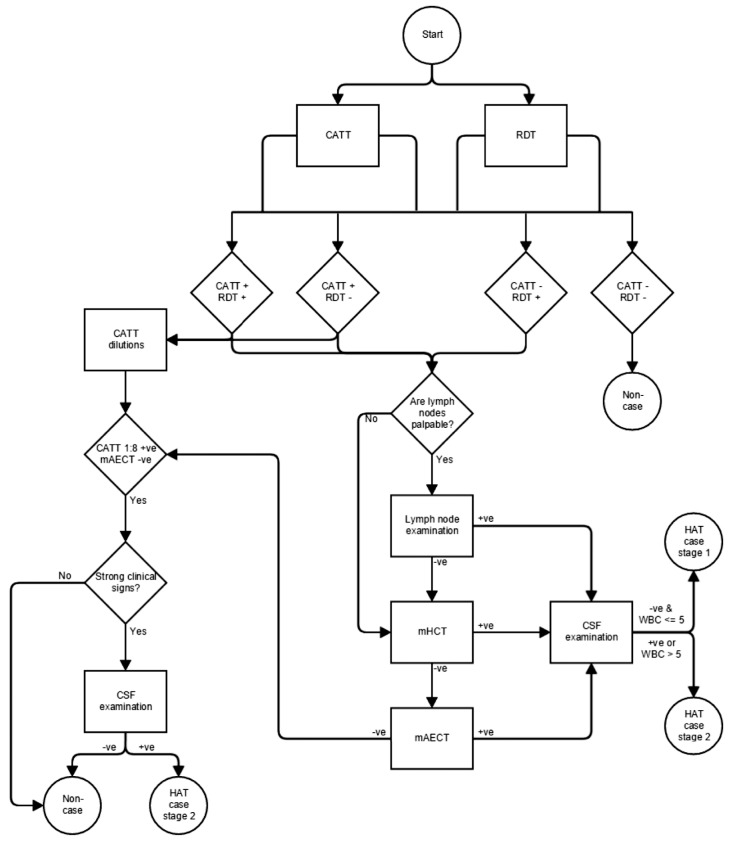
Flow diagram of the diagnostic algorithm.

If an individual was positive by CATT at 1:8 dilution or had symptoms strongly suggestive of HAT, but was negative by examination of lymph node aspirate, mHCT and mAECT, a lumbar puncture may have been performed and the CSF examined for trypanosomes by microscopy, which was at the discretion of the supervisor (Fig 1). Any patients with parasites or more than 5 WBCs per μl in the CSF were interpreted as stage 2 cases. All confirmed HAT cases were treated according to the guidelines of the national programme.

### Data analysis

The definition of a HAT case was a study participant from whom trypanosomes were visualised in any fluid, including a lymph node aspirate, blood or CSF. A control was defined as either an individual found to be both RDT and CATT negative and who had no history of HAT (not treated for HAT in the past), or an individual who was either RDT and/or CATT positive but from whom no trypanosomes were seen in any body fluid (using at least both mHCT and mAECT), and had no history of HAT.

We assessed the sensitivity and specificity of three tests:

- Screening with CATT on whole blood,
- Screening with CATT on whole blood followed by CATT at 1:8 dilution.
- Screening with RDT.

The reference standard was parasitological confirmation using the CRS, and confirmed cases were staged by examination of the CSF.

The results obtained from fixed health facilities and from mobile teams were analysed separately. This is because these are two very different settings–mobile teams operate outdoors and screen all persons that present, whilst fixed facilities only screen clinical suspects who present themselves and high risk individuals. In the analysis, we present the overall RDT result and the results of the individual bands.

### Statistical analysis

Sensitivity was calculated as the number of cases that were positive by a screening test, divided by the total number of cases. Specificity was calculated as the number of controls that were negative by a screening test, divided by the total number of controls. As each screening test result was read by two people, the final number of positive results was the average result from the two readers, and in the event of discordant results, this was included as a 0.5 in the numerator for sensitivity and specificity calculations as per [18]. Results from parasitology were only recorded once, the single result that was confirmed by the supervisor. Patients that were positive by a screening test and were not positive by any parasitological method and had not completed the minimum CRS of mHCT and mAECT were excluded. 95% confidence intervals (CIs) were calculated using the Wilson method implemented in the Hmisc package [22] in the R statistical environment [23]. To test agreement between readers, we calculated Cohen’s Kappa using the fmsb package [24] in R, we interpreted a Kappa of greater than 0.8 as very strong agreement [25] and used the p-value to evaluate whether it is significantly different from zero.

To interpret the impact of test sensitivity and specificity in terms of false positives and false negatives in consideration of the disease prevalence, we calculated the positive predictive value (PPV) as follows:

$$PPV=\frac{sensitivity\;x\;prevalence}{sensitivity\;x\;prevalence+(1-specificity)\;x\;(1-prevalence)}$$

and the negative predictive value (NPV) as follows:

$$NPV=\frac{specificity\;x\;(1-prevalence)}{\left(1-sensitivity)\;x\;prevalence+specificity\;x\;(1-prevalence\right)}$$

To make this a statistic that can be easily translated by a surveillance programme, we present the false discovery rate (FDR) and false omission rate (FOR) as:

FDR=1−PPV

FOR=1−NPV

and we present the FDR as false positives per 100 positive tests and FOR as false negatives per 100 negative tests for prevalences ranging from 0 to 2%.

### Ethics approval and consent to participate

The protocol for this study was approved by the Ethical Review Committee of Ngaliema Clinic, Ministry of Public Health of the Democratic Republic of the Congo (approval number 184/2013). Written informed consent was obtained from all study participants with a positive screening test.

## Results

### Enrolment of participants

**Fig 2:**
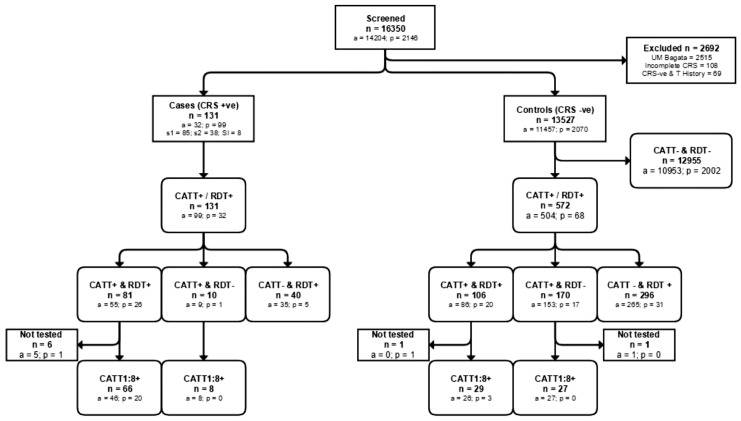
Flow diagram of participant enrolment. Note that for illustrative purposes in this flow diagram, if either reader recorded a positive result then it is recorded as positive in this Figure.

Enrolment of participants was carried out over a period of 7 months, between 14 March 2013 and 7 October 2013 and the average duration of enrolment per site was 3 months. A total of 131 HAT cases were enrolled (99 through active screening and 32 through passive screening). Thirty-eight cases were in the second stage of disease, 85 were in stage 1. A lumbar puncture and CSF examination was not done on 8 confirmed cases, which for analysis, have been interpreted as stage 1 (Fig 2). Cases were diagnosed by gland puncture, mHCT and mAECT in almost equal proportions, while 4 cases were only positive by CSF examination.

The number of controls enrolled was 13,527, of whom 11,457 were through active screening and 2,070 through passive screening. Due to errors in recording of the screening results of sero-suspects that were not confirmed by parasitology by one mobile team, 2,515 potential controls that were enrolled by that mobile team were excluded (Fig 2). Another 108 could not be considered as controls because they were positive by CATT and/or the RDT, but either mHCT or mAECT were not performed, while 65 were excluded because they had a history of HAT.

### Test sensitivity

**Fig 3:**
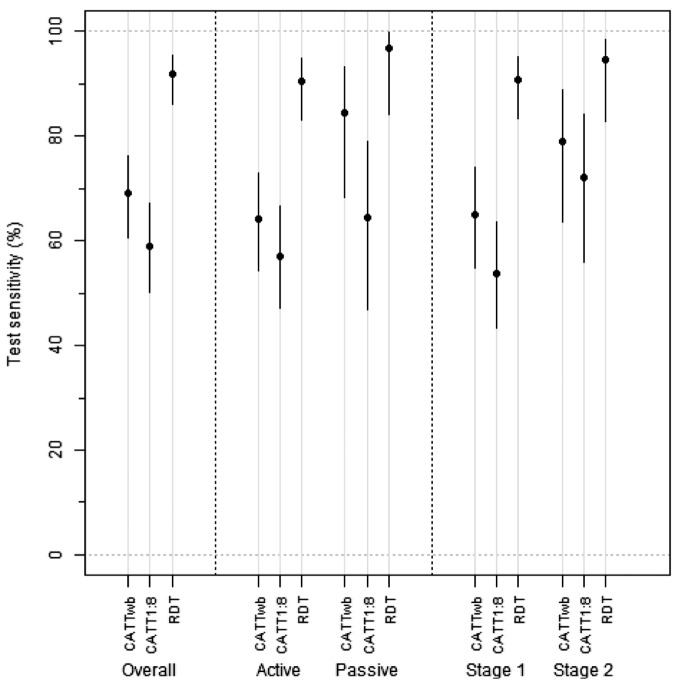
Sensitivity of the tests. Points represent the estimates and lines the 95% CIs. Active and passive refer to active and passive screening.

The sensitivity of the RDT was 92.0% (95% CI = 86.1–95.5%), with band 2 (LiTat 1.5) recording a higher sensitivity than band 1 (LiTat 1.3) (Table 1). CATT on whole blood had a significantly lower sensitivity than the RDT (69.1%; 95% CI = 60.7–76.4%). CATT on plasma diluted 1:8 had a sensitivity of 59.0% (95% CI = 50.2–67.2%). There was significant agreement between readers on all tests (Table 1), although for the RDT, the degree of disagreement between readers was higher on the individual bands than for the test as a whole. The RDT followed by CRS had the highest sensitivity in both mobile teams and fixed facilities, and among stage 1 and stage 2 HAT patients (Fig 3). The performance of the index test in different sites was variable (S1 File) and if the site that performed worst with CATT (Bagata mobile team) is removed from the analysis, then the sensitivity of CATT improves to 77.8% (95% CI = 67.6–85.5%), but remains significantly less sensitive than the RDT (Chi-square p = 0.006).

**Table 1:** Screening test sensitivity.

Test	Reader disagreement (%)	Cohen’s Kappa (p-value)	Pos / N	Sensitivity (%) (95% CI)
RDT	0.8	0.948 (<0.001)	120.5 / 131	92.0 (86.1–95.5)
RDT band 1 (LiTat 1.3)	5.3	0.854 (<0.001)	99.5 / 131	76.0 (68.0–82.5)
RDT band 2 (LiTat 1.5)	3.8	0.861 (<0.001)	109.5 / 131	83.6 (76.3–89.0)
CATT whole blood	0.8	0.982 (<0.001)	90.5 / 131	69.1 (60.7–76.4)
CATT 1:8*	0	1 (<0.001)	74 / 125.5	59.0 (50.2–67.2)

Sensitivity of individual screening tests and agreement between readers as implemented in the field.

* 5 cases that were positive by CATT on whole blood by both readers, and 1 that was positive by reader 1, were not tested using CATT dilutions and were excluded from the section on agreement. For the sensitivity calculations, the denominator is 125.5, because one case had discordant results by CATT on whole blood for the two readers, and CATT dilution was not done on the case. As this case was negative by CATT whole blood by reader 2, it was a complete result for reader 2, but not for reader 1 as dilutions were not performed, and so we counted it as 0.5 of a case in the denominator.

### Test specificity

**Fig 4:**
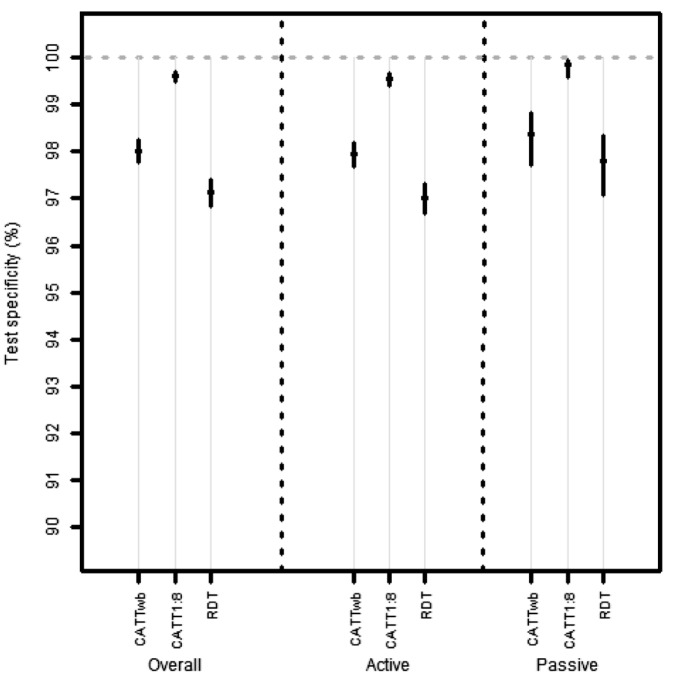
Specificity of the tests. Points represent the estimates and lines the 95% CIs. Note the y-axis range is 90–100%. Active and passive refer to active and passive screening.

The specificity of all tests was greater than 97% and the inter-reader agreement was very good for all the tests (Table 2). The highest specificity was observed with CATT at 1:8 dilution, which was significantly greater than the specificity using either CATT on whole blood or the RDT (Table 2) and this did not vary between active and passive screening (Fig 4). There was relatively little variation in the performance of the index test in different sites in terms of specificity (S1 File).

**Table 2:** Screening test specificity.

Test	Reader disagreement (%)	Cohen’s Kappa (p-value)	Neg / N	Specificity (%) (95% CI)
RDT	0.2	0.964 (<0.001)	13138.5 / 13527	97.1 (96.8–97.4)
RDT band 1 (LiTat 1.3)	0.3	0.934 (<0.001)	13256.5 / 13527	98.0 (97.8–98.2)
RDT band 2 (LiTat 1.5)	0.3	0.925 (<0.001)	13233.5 / 13527	97.8 (97.6–98.1)
CATT whole blood	0.1	0.970 (<0.001)	13259 / 13527	98.0 (97.8–98.2)
CATT 1:8*	0.01	0.990 (<0.001)	13470 / 13525	99.6 (99.5–99.7)

Specificity of individual screening tests and agreement between readers.

* Includes CATT whole blood negatives; 2 suspects who were positive by CATT on whole blood and were not subsequently tested by CATT dilutions were excluded.

### Field application

**Fig 5:**
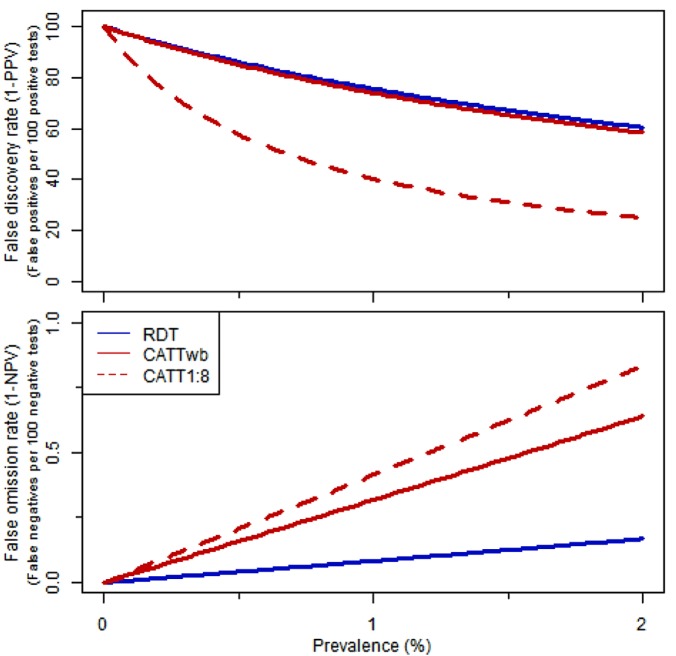
False discovery and false omission rates. FDR and FOR of each test followed by CRS over a range of prevalences, which were calculated based on the sensitivity and specificity of the algorithms that were found in this study.

In the field, at a 1% prevalence, 75.6% of RDT positives would be false positives, compared to 40.3% of positives found by CATT and CATT dilutions. The corollary of this is that for every 10,000 negative screening results (corresponding to approximately 40 active screening days), 8.3 would be false negatives (missed cases) by RDT, compared to 31.8 false negatives by CATT alone (Fig 5).

### Agreement between tests

Approximately 50% of the HAT cases were positive by CATT and by both bands on the RDT (Table 3). This proportion was higher among stage 2 cases, 60–63% of whom were positive by CATT and both bands on the RDT, compared to 44–46% of stage 1 cases, although this difference was not statistically significant (Chi square p > 0.1). When the average of both readers was considered, 72.3% of the stage 2 patients were positive by both bands on the RDT, compared to 66.7% among stage 1 patients (Table 3).

**Table 3:** Combinations of positive screening tests.

	Reader 1			Reader 2*		
	All (%)	Stage 1 (%)	Stage 2 (%)	All (%)	Stage 1 (%)	Stage 2 (%)
CATT & RDTB1 & RDTB2	67 (51.1)	43 (46.2)	24 (63.2)	63 (48.8)	40 (44.0)	23 (60.5)
CATT & RDTB1	4 (3.1)	2 (2.2)	2 (5.3)	5 (3.9)	3 (3.3)	2 (5.3)
CATT & RDTB2	10 (7.6)	8 (8.6)	2 (5.2)	12 (9.3)	9 (9.9)	3 (7.9)
RDTB1 & RDTB2	25 (19.1)	21 (22.6)	4 (10.5)	24 (18.6)	20 (22.0)	4 (10.5)
CATT	10 (7.6)	8 (8.6)	2 (5.3)	10 (7.8)	8 (8.8)	2 (5.3)
RDTB1	6 (4.6)	5 (5.4)	1 (2.6)	5 (3.9)	3 (3.3)	2 (5.3)
RDTB2	9 (6.9)	6 (6.5)	3 (7.9)	9 (7.0)	7 (7.7)	2 (5.3)
All negative	0	0	0	1 (0.8)	1 (1.1)	0
**Total**	**131**	**93**	**38**	**129**	**91**	**38**

Combinations of positive screening tests for the 131 HAT cases that were identified during this study.

RDTB1 = RDT band 1; RDTB2 = RDT band 2.

* Two participants from reader 2 were excluded as the results of the qualitative assessment of band intensity were incomplete

### RDT band intensity

The following observations were made on the qualitative assessment of the intensity of RDT bands:

- The intensity of RDT bands was stronger for HAT cases than for false positive suspects. The mean score on band 1 (LiTat 1.3) was 1.54 for cases, and 1.27 for false positive suspects, which was significantly lower (Wilcoxon rank sum test p = 0.003). The mean score on band 2 (LiTat 1.5) was 1.68 for cases and 1.31 for false positive suspects, which was also statistically significant (Wilcoxon rank sum test p < 0.001).
- When band intensity scores of 1 were considered as negative, the sensitivity of the RDT decreased to 77.5% (95% CI = 69.6–83.8%) and the specificity increased to 97.9% (95% CI = 97.7–98.2%) and there was no significant difference with CATT for either sensitivity (Chi sq p = 0.16) or specificity (Chi sq p = 1).
- There was good agreement between readers on cases and false positive suspects. Both readers gave the same score for band 1 (LiTat 1.3) in 80.2% of cases, and for band 2 (LiTat 1.5) in 90.8% of cases. Among the false positive suspects, the agreement was slightly lower, at 75.9% and 77.6% respectively.
- The intensity score of both bands was the same among 54.2% of the cases for reader 1 and 49.6% of the cases for reader 2, compared to 24.6% and 29.9% for false positive suspects.
- Among the 402 identified as false positive suspects by the RDT, the largest proportion (16.2% and 17.1%) had an intensity score of 2 on band 2 (LiTat 1.5) and 0 on band 1 (LiTat 1.3) by the two readers. Among cases, 31.3% and 29.8% had an intensity score of 2 on each band for each reader.

## Discussion

This study has demonstrated that the SD BIOLINE^®^ HAT RDT had a higher sensitivity in both active and passive screening, with a difference of 23% between it and the second best screening test—CATT (Table 1 and Fig 3). However, this is at the expense of a slightly lower specificity of the RDT, which would result in some additional workload in confirmatory testing. The simplicity and stability of the HAT RDT has created a great opportunity to improve screening coverage of the population at risk, as it can be deployed to any health facility in endemic areas. There were no issues regarding reproducibility–the agreement between readers was very good for both CATT and the RDT. However, there was a case that would have been missed if there was just one reader for each test, which highlights the importance of training and diligence of staff who are reading the tests.

The genome of *T*. *b*. *gambiense* codes for a large number of VSG antigens that are expressed differentially during the course of infection [26] and it has been reported that the VSGs LiTat 1.3 and LiTat 1.5 are predominantly expressed by *T*.*b*. *gambiense* [27]. The difference in sensitivity that we report here between the RDT and CATT could be due in part to the inclusion of the LiTat 1.5 antigen, and as such it could be assumed that a patient infected with trypanosomes that had expressed only the VSG LiTat 1.5 antigens might be missed by CATT and only detected using the RDT. Interestingly, there were more cases detected by band 2 of the RDT (VSG LiTat 1.5) than by band 1 (VSG LiTat 1.3) and the band 2 antigen was responsible for identifying 19 cases that would have been missed if only the band 1 antigen had been used in the RDT. Similar observations were made in a clinical trial on the prototype of the same RDT [21]. An explanation for this could be that widespread and continuous use of CATT as the main screening tool for several decades could have resulted in a strong selection pressure against parasites expressing LiTat 1.3 antigens. Previous studies have reported that some sub-populations of *T*.*b*. *gambiense* did not harbour the LiTat 1.3 gene [28], which would prevent detection using CATT. This in turn might explain the relatively low sensitivity of the CATT test that was observed in this study, which would not have been identified without using another screening test based on different antigens and different presentation of the antigens to identify cases. Conducting a large study incorporating two screening tests has identified a number of cases that could have been missed in previous studies using CATT alone. It is therefore conceivable that including a third antigen in the RDT could further increase its sensitivity, but may also decrease specificity.

Most HAT cases were positive in both bands of the RDT. This suggests that at some point during infection, HAT cases had waves of parasitaemia with variant antigenic types (VATs) of parasites expressing each of LiTat 1.3 and LiTat 1.5 VSGs, and that the corresponding humoral response was maintained. This may also explain why stage 2 cases were more frequently positive by both RDT bands than stage 1 cases, as they were more likely to be infected for long enough to be exposed to multiple waves of parasitaemia (Table 3).

Another possible explanation for the observed difference in sensitivity between CATT and the RDT could be the difference in the format and chemistry of these tests. While the CATT test is based on the agglutination of freeze-dried fixed and stained trypanosomes with host antibodies, the RDT relies on the formation of a complex made of a nitrocellulose-bound antigen, host antibodies and a gold conjugated antigen. The antigens in the RDT are separated from the trypanosome, exposing other epitopes that in the fixed parasites used in the CATT test would remain hidden, such epitopes would bind other antibodies present in the patient, also contributing to better sensitivity. In addition, the composition of the dilution buffer that is used with the RDT is not publicly known, and it could be that it is different from that used in the CATT test, which could influence antigen-antibody binding.

During this study, CATT was used according to the manufacturer’s instructions and staff performing the test ensured that both positive and negative controls reacted according to instructions and no failure to follow usage or storage protocols were observed. However, it remains possible that the antigen could have deteriorated within the limits of the positive control, resulting in decreased sensitivity, or the possibility of a weak agglutination that was not easily detected by the technicians. If there was deterioration, then this might explain some of the differences in CATT sensitivity between what was observed here and what was observed in a clinical study to evaluate the prototype RDT [21].

To estimate the true sensitivity of the RDT and CATT, it is necessary to identify all cases in the study population. Therefore, a weakness in this study is that it assumes that all cases would test positive with either CATT or RDT, but there could have been people infected with VATs that had not expressed any of the antigens in both tests. However, due to logistical challenges of screening such large numbers of people in a prospective study, it was not possible to perform parasitological confirmation on all subjects screened. As a consequence, subjects were only tested by parasitology if they were positive by RDT or CATT. If we were to assume that both CATT and RDT were independent, with sensitivities of 69.1% and 92.0%, then using both tests in parallel and taking a positive on either test as a serological suspect would give a sensitivity of 97.5%, and as such if the tests were independent, then screening with both tests would result in missing 2.5% of cases. However, as the sensitivities of the two tests are not independent–due to the sharing of an antigen, the true combined sensitivity may be lower than 97.5% and more than 2.5% of cases missed. These missed cases and subsequent over-estimation of the sensitivity of the test is an unavoidable limitation of this study design. Consequently, the results of this analysis can be regarded as the conditional field sensitivity of the tests. Whilst the use of parasitological methods and other reference tests such as immune trypanolysis (TL) would be desirable, these techniques do not have 100% sensitivity, and would therefore still miss some cases [8,29].

In all HAT studies and screening programs, the specificity of the parasitological techniques is assumed to be 100%, but false positives have been reported in the past [30,31]. In this study, all HAT cases that were positive by parasitology were verified by a supervisor, thus minimising the risk of having false positives by parasitology, but there does remain a small risk of over-diagnosis. In terms of sensitivity, there is no reason to assume that false positives by parasitology would alter the comparisons between index tests. Whilst it would be desirable to perform TL as a reference test [8,29], it was not possible to collect samples for TL under this study design, but future studies should make efforts to incorporate TL.

The sensitivity and specificity of both CATT and RDT was higher in passive screening at fixed health facilities than in active screening, albeit not statistically significant. A possible explanation could be that there was a greater proportion of cases in stage 2 that were diagnosed at healthcare facilities. Due to their longer duration of infection, stage 2 cases would be more likely to have mounted an immune response to the VSGs used in these tests, resulting in higher sensitivity. The difference might also be due to sub-optimal blinding in healthcare facilities. Laboratory technicians in healthcare facilities are more likely to be aware of the clinical status of patients, mainly because patients are normally only screened for HAT at healthcare facilities if they have clinical signs, whereas at mobile teams, clinical signs are not considered prior to screening. This difference could have introduced a bias when interpreting the screening test results.

CATT dilutions had considerably greater specificity but lower sensitivity than CATT on whole blood and RDT. When these sensitivities are translated into case detection at the population level, the lower specificity of the RDT or CATT on whole blood during active screening leads to a larger number of suspects having to be taken through parasitological confirmation, which could be expensive and logistically challenging to screening teams. However, the corollary of this is that the improved sensitivity of the RDT followed by parasitological confirmation means that at 1% prevalence, there are around four times fewer false negative cases (missed cases) among those that test negative by the RDT than by the next most sensitive test (CATT). A balance would need to be struck in terms of positive and negative predictive values, which should be evaluated in a cost-benefit analysis for various prevalence values. From a practical perspective, a mobile team usually screens between 200 and 300 people in one day. In a setting where the prevalence of HAT would be around 0.8%, an RDT with a specificity of 97% would detect a mean of 2 cases and 8 false positives per day. Although such a number is not large for a mobile team, the extra burden placed on the team and costs associated with testing the false positive suspects should be established.

This study was limited by the relatively small number of cases that were identified. Whilst the regions of the DRC that were selected for the study are among the most endemic, the prevalence of HAT in many parts of Africa has been falling, as was reflected in the prevalence of less than 1% among those that were enrolled in this study. A larger number of cases would have enabled us to get a more precise estimate of the sensitivity of the tests. With declining prevalence and corresponding decrease in positive predictive values, it could soon be necessary to develop diagnostics with even higher accuracy, to minimise the number of cases that are missed and achieve elimination, whilst minimising the workload and cost.

## Conclusions

This study has demonstrated that the SD BIOLINE^®^ HAT RDT has superior sensitivity when screening for *gambiense* HAT in both active and passive screening settings in the DRC. However, it still misses at least 8% of the cases, meaning that there remains scope for developing other screening tests with better performance. Other test combinations that were tested here offer better specificity, which would require further investigations in cost-effectiveness analysis to determine the optimal combination, especially for accelerated and sustained elimination of the disease. The RDT would also benefit from further testing in other settings. Serological tests with improved accuracy, if they can be developed, could then be used as true diagnostic tests, without the need for confirmation, and might also be used for the identification of asymptomatic carriers of HAT [32]. In the event that drugs that are safer and easier to use become available [33–35], a "test and treat" strategy would be feasible [36], thus accelerating elimination of the disease.

## Supporting information

S1 File Additional analysis. Additional analysis not included in the manuscript file.
(PDF)

S2 File Study protocol. The study protocol that was followed in the field.
(PDF)

S3 File Study data. (XLSX)

S1 Table STARD checklist. STARD checklist for the reporting of studies of diagnostic accuracy.
(PDF)
