# Supplementary material for: Performance of the SD BIOLINE® HAT rapid test in various diagnostic algorithms for gambiense human African trypanosomiasis in the Democratic Republic of the Congo
Source: PLoS One. 2017 Jul 3;12(7):e0180555. doi: 10.1371/journal.pone.0180555 (PMC5495481; doi:10.1371/journal.pone.0180555)
Supplement: S1 File — Additional analysis not included in the manuscript file. (PDF) [file pone.0180555.s001.pdf]

Supplementary analysis information for **Performance of the SD BIOLINE® HAT rapid test in various diagnostic algorithms for *gambiense* human African trypanosomiasis in the Democratic Republic of the Congo**

Crispin Lumbala, Paul R. Bessell, Pascal Lutumba, Sylvain Baloji, Sylvain Biéler, Joseph M. Ndung'u

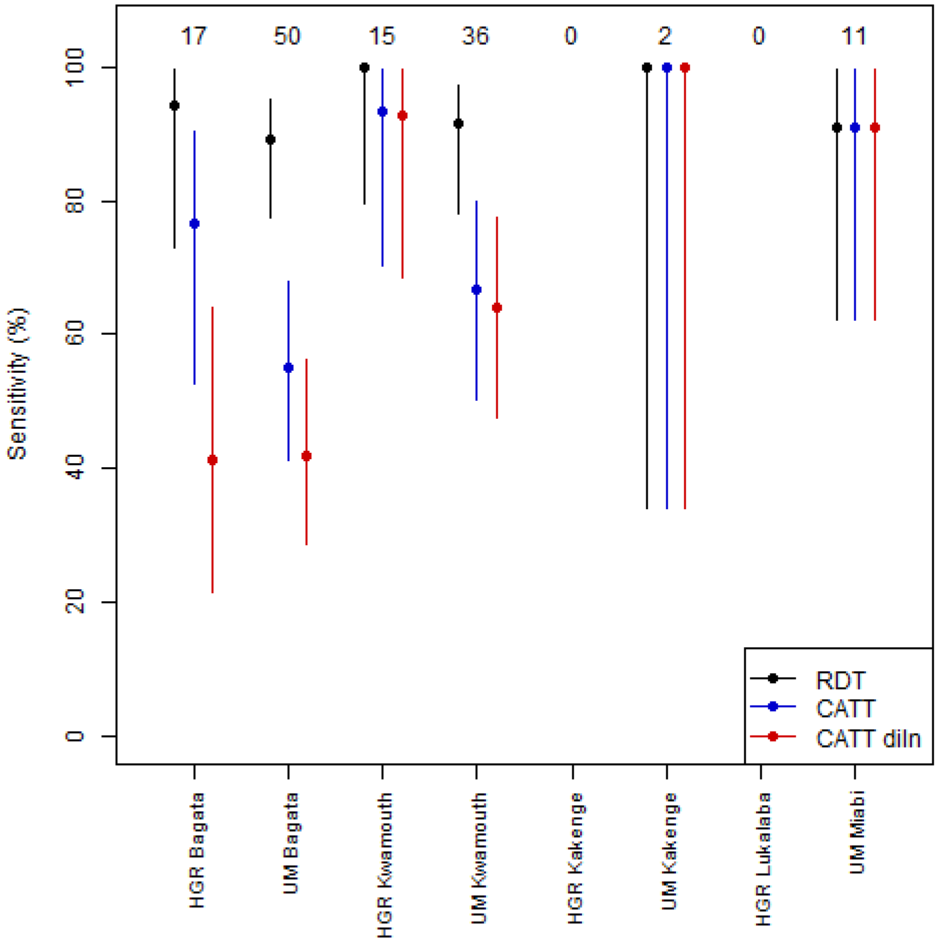

Fig S1. Sensitivity by screening facility. Lines represent the 95% CIs. Numbers at the top are the number screened in each group. HGR = Reference hospital; UM = Mobile team.

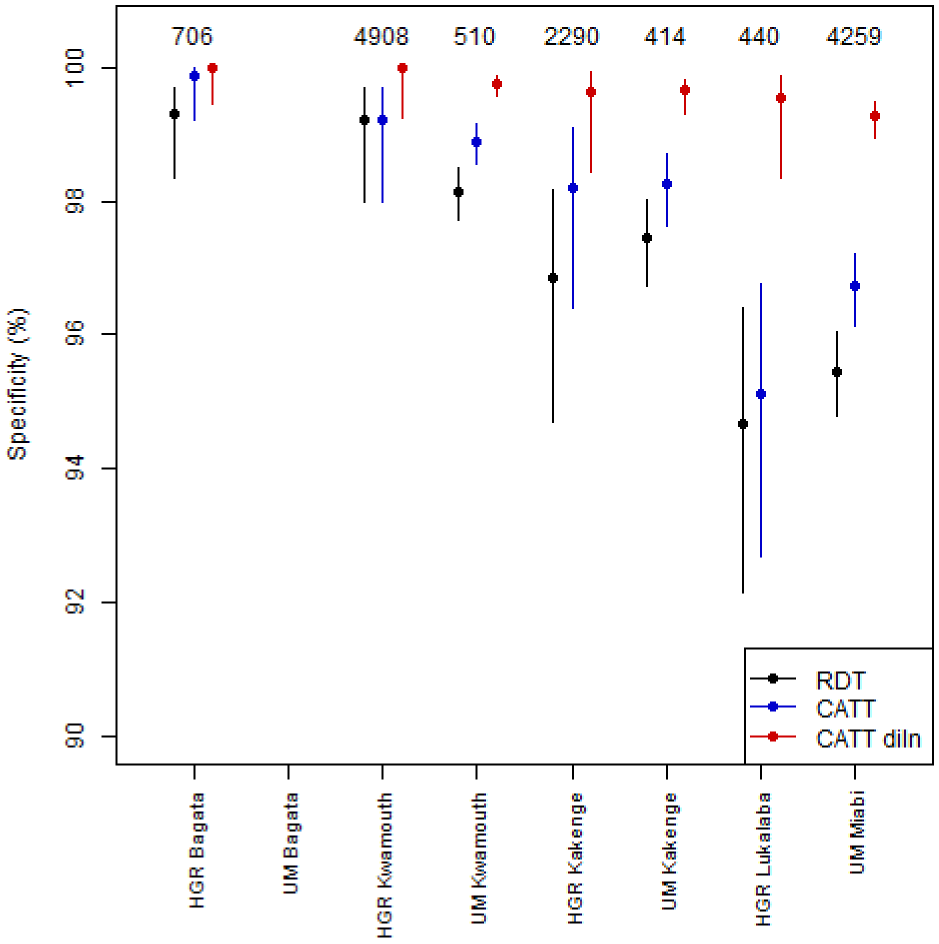

Fig S2. Specificity by screening facility. Lines represent the 95% CIs. Numbers at the top are the number screened in each group. Note the x-axis scale is 0-90%. HGR = Reference hospital; UM = Mobile team.

		Reader 1- borderline		Reader 2 - borderline	
	Number of cases	CATT Negative	Positive	Negative	Positive
Bagata Hospital	17	0	4	0	5
Bagata Mobile team	50	5	4	6	6
Kwamouth Hospital	15	0	0	0	0
Kwamouth Mobile team	36	0	0	0	0
Kakenge Hospital	0	0	0	0	0
Kakenge Mobile team	2	0	0	0	0
Lukalaba Hospital	0	0	0	0	0
Miabi Mobile team	11	0	0	0	0

Table S1. Borderline CATT results amongst cases by unit screening.
