# Supplementary material for: Performance of the SD BIOLINE® HAT rapid test in various diagnostic algorithms for gambiense human African trypanosomiasis in the Democratic Republic of the Congo
Source: PLoS One. 2017 Jul 3;12(7):e0180555. doi: 10.1371/journal.pone.0180555 (PMC5495481; doi:10.1371/journal.pone.0180555)
Supplement: S2 File — The study protocol that was followed in the field. (PDF) [file pone.0180555.s002.pdf]

FIND
foundation
for innovative new diagnostics

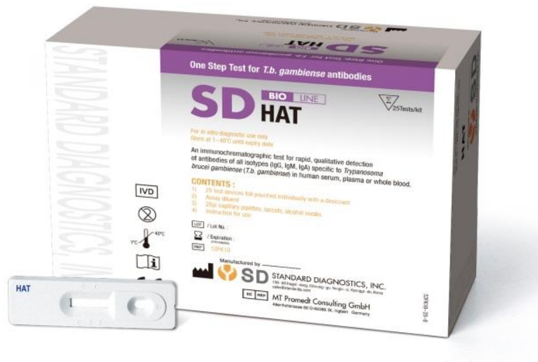

STUDY PROTOCOL

SD HAT RDT demonstration study (Democratic Republic of the Congo)

Demonstration study of a rapid diagnostic test for detection of antibodies against *Trypanosoma brucei gambiense* in human blood in the Democratic Republic of the Congo

Date: 8th February 2013

Project: HAT Antibody Detection #7730

Study number: 7730-3-1

Study sites: Kwamouth and Bagata reference hospitals and Kwamouth and Bagata mobile teams in Bandundu province, Lukabala reference hospital and Miabi mobile team in East Kasaï province, Kakenge health centre and Kakenge mobile team in West Kasaï province.

Principal Investigator

Dr Crispin Lumbala, PNLTHA, DRC
Email: doccrislumbala@yahoo.fr
Tel: +243 99 002 7220

Study Coordinator

Dr Joseph Ndung'u, FIND
Email: joseph.ndungu@finddiagnostics.org
Tel: +41 22 710 93 11

Project Manager

Dr Sylvain Biéler, FIND
Email: sylvain.bieler@finddiagnostics.org
Tel: +41 22 710 27 81

Confidentiality statement

The information contained in this document, especially unpublished data, is the property of FIND (or under its control) and may not be reproduced, published or disclosed to others without written authorization.

Abbreviations

CAR	Central African Republic
CATT	Card Agglutination Test for Trypanosomiasis
CRF	Case Report Form
CRS	Composite Reference Standard
CSF	Cerebrospinal fluid
CTC	Capillary Tube Centrifugation
DRC	Democratic Republic of the Congo
FIND	Foundation for Innovative New Diagnostics
GP	Gland Puncture
HAT	Human African Trypanosomiasis
mAECT	mini Anion Exchange Centrifugation Technique
PI	Principal Investigator
PNLTHA	Programme National de Lutte contre la Trypanosomiase Humaine Africaine
RDT	Rapid Diagnostic Test
SD	Standard Diagnostics
T.b.	*Trypanosoma brucei*
WHO	World Health Organization

Statement of principal investigator

In signing this page, I agree to conduct the study in accordance with the relevant, current protocol, applicable regulations and institutional policy.

I will ensure that the requirements relating to obtaining institutional review board (IRB) review and approval are met. I will promptly report to the IRB all changes in the research activity.

I agree to notify my FIND colleagues and the co-investigators prior to making any changes in the protocol.

I will maintain confidentiality of protocol and investigational materials.

I agree to ensure that all colleagues and employees assisting in the conduct of the study are informed about their obligations in meeting the above requirements.

____

[Dr Crispin Lumbala, PNLTHA, DRC] Date

TABLE OF CONTENTS

1. STUDY OVERVIEW AND PROTOCOL SUMMARY 5

2. INTRODUCTION AND CONTEXT...5

3. STUDY OBJECTIVES..6

4. HYPOTHESES (FOR OBJECTIVE 1) 6

5. STUDY DESIGN (FOR OBJECTIVE 1)..8

6. STUDY ENDPOINTS..8

7. STUDY SITES AND ENROLMENT OR PARTICIPANTS...8

8. COMPOSITE REFERENCE STANDARD 9

9. DEFINITIONS OF CASES AND CONTROLS 9

10. SAMPLE SIZE CALCULATION 10

11. STUDY WORKFLOW AND PROCEDURES 10

11.1 Diagnostic procedures...10
11.2 Management of patients with positive test results...12
11.3 Follow-up 12
11.4 Cost analysis..12
11.5 Cost effectiveness..13
11.6 Ease of implementation..13

12. BLINDING 13

13. ETHICAL CONSIDERATIONS...14

14. EQUIPMENT AND REAGENT NEEDS 14

15. DATA MANAGEMENT AND ANALYSIS 14

16. MINIMIZATION OF BIAS 15

17. QUALITY ASSURANCE AND QUALITY CONTROL...15

18. TRIAL SITE ASSESSMENT..15

19. STUDY COORDINATION, TRAINING AND MONITORING 15

20. DISSEMINATION OF RESULTS AND PUBLICATION POLICY.................................16

21. RISKS AND MITIGATION STEPS 16

22. REFERENCES...16

1. Study overview and protocol summary

Study objective: To determine the performance of various diagnostic strategies including the use of the RDT and/or CATT, determine the cost of using the RDT and/or CATT as part of these strategies, determine the cost effectiveness of diagnosing and treating patients with these strategies, and assess the ease of implementation of these diagnostic strategies.

Study population: 1) Individuals screened actively by mobile teams.
2) Individuals presenting themselves at health centres/reference hospitals and who are screened passively.

Study sites: Kwamouth and Bagata reference hospitals and Kwamouth and Bagata mobile teams in Bandundu province, Lukabala reference hospital and Miabi mobile team in East Kasaï province, Kakenge health centre and Kakenge mobile team in West Kasaï province.

Sample size: At least 242 parasitologically confirmed HAT cases (active and passive enrolment combined), 1,500 passively enrolled controls and 15,000 actively enrolled controls.

Study duration: 19 weeks, including enrolment of participants for 12 weeks.

Study timeline:

Activity	1	2	3	4-5	6	7	8-9	10	11	12	13-15	16	17	18	19
Study site preparation	■														
Training		■	■												
Study site assessment			■												
Participant enrolment				■	■	■	■	■	■	■	■				
Data entry					■			■				■			
Monitoring visits			■			■			■				■		
Data analysis							■			■				■	
Report writing															■

2. Introduction and context

Human African trypanosomiasis (HAT) is a neglected tropical disease that is endemic in 36 countries of sub-Saharan Africa. Various HAT diagnostic algorithms include successive screening, parasitological confirmation and staging steps (Checchi *et al.*, 2011). Thanks to

international and national efforts, the number of cases reported to WHO have been declining (Simarro *et al.*, 2011). However, a reduction in vector control or surveillance activities could lead to re-emergence of the disease (Chappuis *et al.*, 2010). Thus, novel strategies should be implemented to accelerate control and/or elimination of the disease. Success of such strategies is hampered by difficulties in diagnosis of the disease as current techniques are not sensitive enough, and not easy to use in peripheral health centres or general hospitals. Indeed, HAT patients can stay long before the correct diagnosis is made, ending up in the advanced or second stage of the disease. Second stage disease is more difficult and expensive to manage (drugs and hospital stay). There is therefore an urgent need for an accurate, easy to use and affordable test for HAT (Sterveding, 2006; Kennedy 2008, Debborggraeve *et al.*, 2010). The test should enable better integration of HAT diagnosis in the general primary health care system. The Foundation for Innovative New Diagnostics (FIND) and Standard Diagnostics, Inc. (SD) have developed a rapid diagnostic test (RDT) (SD BIOLINE HAT) for HAT and compared its performance with the Card Agglutination Test for Trypanosomiasis (CATT). Initial laboratory studies, which were carried out on a prototype RDT at the International Livestock Research Institute (ILRI), Kenya, and Institute of Tropical Neurology (INT) in France using stored plasma samples from parasitologically confirmed HAT patients and negative controls, yielded promising results. These were followed by clinical trials to compare the performance of the RDT to that of CATT, which were conducted in Angola, Democratic Republic of the Congo (DRC) and Central African Republic (CAR) on more than 14,000 participants, and which confirmed the good performance of the RDT, with an overall test sensitivity of 89.3% (95% CI: 83.3-93.3) and specificity of 94.6% (95% CI: 94.2-94.9). Based on these results, the design of the product was locked, the RDT was CE-marked and registered in the Republic of Korea, and officially launched in December 2012. A demonstration study will now be conducted in multiple sites in DRC to (i) determine the performance of various diagnostic strategies including the use of the RDT and/or CATT, (ii) determine the cost of using the RDT and/or CATT as part of various diagnostic strategies, (iii) determine the cost effectiveness of diagnosing and treating patients with these strategies and (iv) assess the ease of implementation of these diagnostic strategies. This study is expected to provide additional evidence on the performance of the RDT as part of a diagnostic algorithm, as well as financial and operational information that will allow determination of its suitability for introduction into programmatic use.

3. Study objectives

The main objectives of this study are:

Objective 1: To determine the performance of various diagnostic algorithms including the use of the SD BIOLINE HAT test and/or the CATT test to diagnose *T. b. gambiense* HAT.

Objective 2: To determine the total cost of using the RDT and/or the CATT test as part of various diagnostic algorithms.

Objective 3: To determine the cost effectiveness of diagnosing and treating HAT patients using various diagnostic algorithms including the RDT and/or the CATT test.

Objective 4: To assess the ease of implementation of the RDT and of CATT in various diagnostic strategies.

4. Hypotheses (for Objective 1)

Four different diagnostic algorithms will be evaluated in this study, as described in Figure 1 below. Suspects will be first identified using either CATT or the RDT performed on a sample of whole blood obtained from a finger prick, followed or not by a CATT test performed on

serially diluted plasma, and finally confirmed by parasitology (CRS, composite reference standard, see Point 8 below) before staging and treatment. A cut-off of 1:8 will be used for the CATT dilution test. Note that these 4 diagnostic algorithms will not be followed in practice during the study; instead, their performance will be calculated retrospectively based on the data collected in the study. The procedures that will be followed with study participants are different from these algorithms and are described in Figure 2 below (both the RDT and CATT will be performed on all participants).

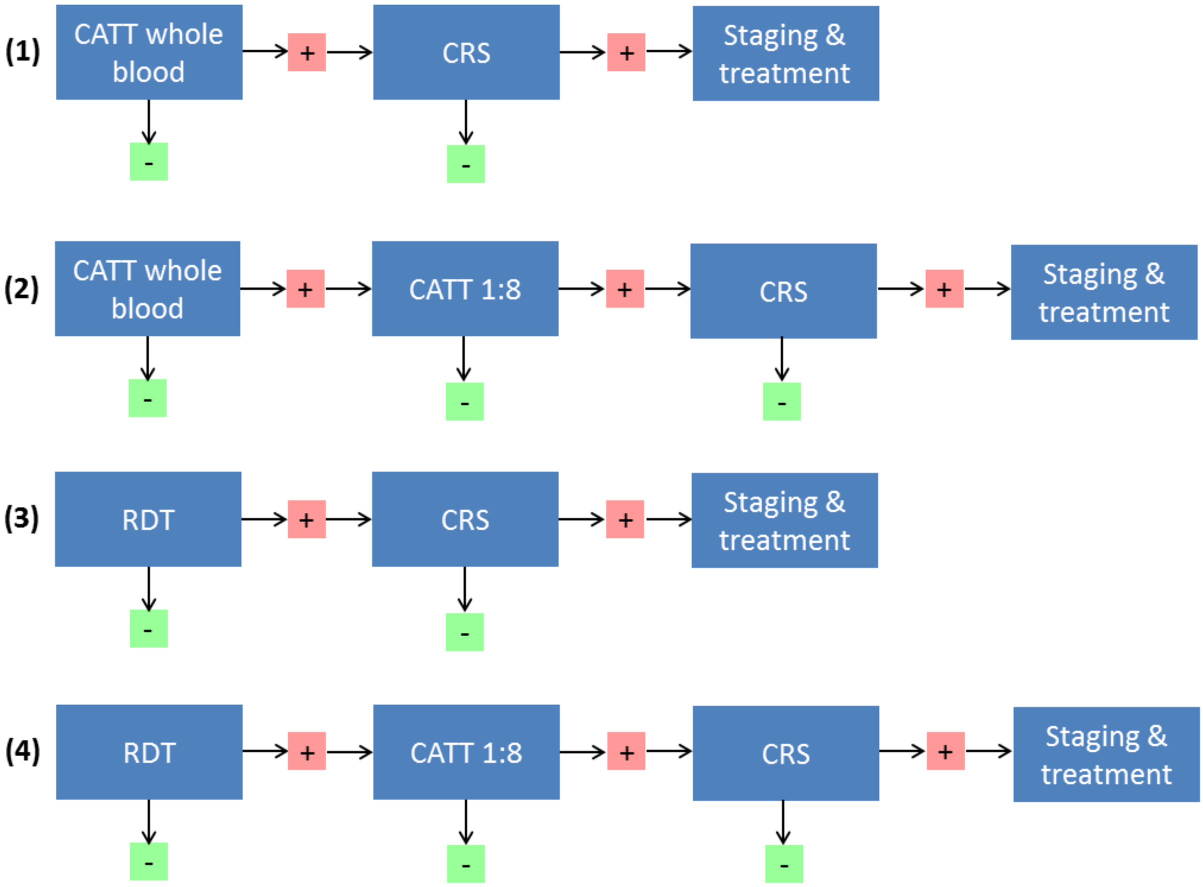

Figure 1. *The 4 diagnostic algorithms that will be evaluated in the study.*

Null hypothesis 1: Detection of HAT cases using diagnostic algorithm 3 is less sensitive than using diagnostic algorithm 2, HAT being defined by the CRS. The non-inferiority margin is set at 7%.

H_{01}: SE_{ALG2} – $SELCI95\%_{ALG3} \geq 7$ %

where SE_{ALG2} is the sensitivity of diagnostic algorithm 2 and $SELCI95\%_{ALG3}$ is the lower limit of the 95% confidence interval of the sensitivity of diagnostic algorithm 3.

Alternative hypothesis 1: Detection of HAT cases using diagnostic algorithm 3 is as sensitive as using diagnostic algorithm 2.

H_{A1}: SE_{ALG2} – $SELCI95\%_{ALG3} < 7$ %

Null hypothesis 2: The SD BIOLINE HAT test performed on fresh blood obtained by finger prick is less specific to diagnose HAT than the CATT test performed on whole blood, HAT being defined by the composite reference standard. The non-inferiority margin is set at 5%.

H_{02}: $SP_{CATT} – SPLCI95\%_{RDT} \geq 5$ %

where SP_{CATT} is the specificity of the CATT test performed on whole blood and $SPLCI95\%_{RDT}$ is the lower limit of the 95% confidence interval of the specificity of the SD BIOLINE HAT test performed on whole blood.

Alternative hypothesis 2: The SD BIOLINE HAT test is as specific as the CATT test performed on whole blood.

H_{A2}: $SP_{CATT} – SPLCI95\%_{RDT} < 5$ %

5. Study design (for Objective 1)

This will be a non-inferiority study in which the sensitivity of HAT case detection using a diagnostic algorithm including the use of the RDT (algorithm 3) will be evaluated in comparison to the sensitivity of another diagnostic algorithm including the use of the CATT test performed on whole blood followed by CATT dilutions (algorithm 2), and in which the diagnostic specificity of the RDT will be evaluated in comparison to the CATT test performed on whole blood. Sensitivity and specificity will be calculated using a composite reference standard (see Point 8).

6. Study endpoints

The study endpoints for each study objective will be as follows:

Objective 1

- Sensitivity of HAT case detection using diagnostic algorithms 1, 2, 3 and 4 in active and in passive screening
- Specificity of the CATT test performed on whole blood as part of diagnostic algorithm 1, specificity of the CATT test performed on plasma diluted 1:8 as part of diagnostic algorithm 2, specificity of the RDT performed on whole blood as part of diagnostic algorithm 3 and specificity of the CATT test performed on plasma diluted 1:8 as part of diagnostic algorithm 4, in active and in passive screening
- Inter-reader reproducibility of the RDT and the CATT test.

Objective 2

- Total cost of implementing and using diagnostic algorithms 1, 2, 3 and 4 in active screening, per population screened
- Total cost of implementing and using diagnostic algorithms 1, 2, 3 and 4 in passive screening, per population screened

Objective 3

- Cost effectiveness of diagnosing and treating HAT patients using diagnostic algorithms 1, 2, 3 and 4 in active screening
- Cost effectiveness of diagnosing and treating HAT patients using diagnostic algorithms 1, 2, 3 and 4 in passive screening

Objective 4

- Ease of implementation of diagnostic algorithms 1, 2, 3 and 4 in active screening
- Ease of implementation of diagnostic algorithms 1, 2, 3 and 4 in passive screening

7. Study sites and enrolment or participants

Enrolment of participants will be done passively at 4 health centres and actively by 4 mobile teams:

- Kwamouth and Bagata reference hospitals and Kwamouth and Bagata mobile teams in Bandundu province
- Lukabala reference hospital and Miabi mobile team in East Kasaï province
- Kakenge health centre and Kakenge mobile team in West Kasaï province

Passive enrolment will consist of individuals presenting to health centres because of suspicion of HAT. They will either be referred from primary health centres that are not equipped to perform HAT diagnostics, on the basis of the presence of clinical signs suggestive of HAT, or present themselves directly to the HAT diagnostic centre.

8. Composite reference standard

To determine the accuracy of the various diagnostic algorithms under investigation, a composite reference standard (CRS) will be used, which will be considered positive if any of the routine parasitological reference tests performed will be positive. A wet smear will be prepared from aspirates of the cervical lymph node (if palpable) and examined for motile parasites by bright field microscopy. The capillary tube centrifugation test (CTC) will be performed, and if its result is negative the mAECT will also be performed. The CTC involves collecting blood in a heparinised capillary tube and after centrifugation, examining the buffy coat for motile parasites by bright field microscopy. The mAECT is performed with a commercial kit. The eluate obtained is centrifuged and the sediment examined for motile trypanosomes by bright field microscopy. In a few cases, it is possible to demonstrate trypanosomes in the CSF, even when all the other tests are negative. Such cases will also be included in the study. The CRS will be considered negative if no trypanosomes are found in blood (using CTC and mAECT results), lymph juice (in case of adenomegaly allowing a lymph node puncture) and CSF (if lumbar puncture is performed, as per routine procedures).

9. Definitions of cases and controls

Cases: Participants will be considered HAT cases when the CRS will be positive (see Point 8). In other words, cases will need to have trypanosomes observed in either blood, lymph node aspirate or cerebrospinal fluid.

Controls: Controls will be defined as follows:

- Either: Individuals found to be both RDT and CATT whole blood negative and who have no history of HAT (note that there will be no CRS results available for these individuals)
- Or: Individuals who are either RDT and/or CATT whole blood positive but who have a negative CRS and no history of HAT

Note that symptoms, including neurological signs that may raise suspicion of HAT, will not be considered as exclusion criteria for controls.

Controls will be enrolled both passively and actively. Passive enrolment of controls will be done on individuals presenting at health centres for HAT testing until reaching at least 1,500 controls. Active enrolment of controls will be done by mobile teams until a total of at least 15,000 controls is reached. To ensure that these controls are representative of the 3 different mobile teams involved, each mobile team will enrol at least 4,500 controls.

Individuals presenting at health centres who are found to be negative with both the RDT and CATT but who have clinical signs suggestive of HAT will also be tested by routine

parasitology methods, as per PNLTHA procedures. These patients will be enrolled as HAT cases if they have a positive CRS, whereas they will be enrolled as controls if their CRS is negative and they have no history of HAT. The clinical signs that are suggestive of HAT are defined as follows:

- Speech disorders
- Enlarged cervical glands
- Behavioural disorders
- Walking impairment
- Sleeping disorders
- Convulsion/epilepsy

10. Sample size calculation

Based on the clinical trials that were conducted during development, the expected sensitivity of diagnostic algorithms 2 and 3 would be identical (89.3%). Using a non-inferiority margin of 7%, a confidence level (1-alpha) of 95% and a power (1-beta) of 80%, the number of HAT cases will need to be at least 242 (Blackwelder, 1982). In DRC, the average HAT prevalence has been recently estimated by the national programme to be 1.25% in the target study population. Therefore, since it is planned to screen 19,800 participants during this study, it is expected that approximately 248 HAT cases will be enrolled. In addition to DRC, the possibility to conduct similar studies in other HAT endemic countries will also be explored (e.g. Angola, CAR or South Sudan).

Based on the same trials, it is expected that the specificity of the RDT (94.6%, 95% CI: 94.2-94.9) will be close to that of the CATT test performed on whole blood (95.9%, 95% CI: 95.6-96.3). Using a margin of 5%, confidence level (1-alpha) of 95% and a power (1-beta) of 80%, a minimum of 409 controls would be needed. This will be easily achieved, and in order to obtain even more accurate estimates, at least 15,000 controls will be enrolled by active screening and at least 1,500 controls will be enrolled by passive screening.

**Study group**	**Minimal number of participants**
**HAT cases (active + passive screening)**	**242**
**Controls (active screening)**	**15,000**
**Controls (passive screening)**	**1,500**

Table 1. Minimal number of participants to be enrolled in each study group.

11. Study workflow and procedures

11.1 Diagnostic procedures

The procedures to be carried out on the study participants are summarized in Figure 2. A CATT and an RDT tests will be performed on whole blood obtained by finger-prick for each participant enrolled by active or passive screening. CATT and RDT results will be read by 2 independent readers and the results recorded separately. Based on the CATT and RDT results, 4 categories of participants will be encountered as indicated in Figure 2 and described below. The intensity of the RDT bands (C, 1 and 2) will be recorded using a colour scale provided by the RDT manufacturer. In case of low-intensity RDT results (1+ or trace), the exact intensity percentage will be recorded using the scale. For the CATT test, borderline

results (+/-) will also need to be specified. After informed consent, all individuals included in categories 1, 2 or 3 (as shown in Figure 2 and described below) will have 5 ml venous blood collected in a heparinized tube. 650 µL blood will be used to perform mAECT (350 µL) and CTC (4 capillary tubes of 75 µL). The remaining volume will be centrifuged, from which 100 µL of buffy coat will be spotted on Whatman filter paper for future molecular analysis and 30 µL of plasma will be used to prepare CATT dilutions. Results of CATT dilutions will also be read by two independent readers and results recorded separately. To evaluate a possible prozone effect with the RDT, participants in category 3 who will be found negative with the RDT but positive at dilution 1:8 with CATT will be tested with a second RDT using a plasma sample diluted 1:8 (the same dilution sample as used for CATT titration). All participants in categories 1, 2 and 3 will be tested for malaria using an RDT in active screening, while all participants will be tested with a malaria RDT in the case of passive screening. Those who are positive for malaria will be examined and treated in line with the national guidelines. Buffy coat samples collected on filter paper will be shipped to a reference laboratory for future molecular analysis. Any individual in these 3 categories with palpable cervical glands will have a gland puncture done and examined for parasites. A lumbar puncture will be performed not only on all HAT cases confirmed by gland puncture, CTC or mAECT, but also on all participants from categories 1 and 3 with a CATT 1:8 positive result, as well as on other participants with clinical signs that are strongly suggestive of HAT.

The 4 categories of participants identified at the fixed centres and during active screening as shown in Figure 2 are as follows:

1. CATT whole blood positive (by at least one reader), HAT RDT positive (by at least one reader) subjects

2. CATT whole blood negative, HAT RDT positive (by at least one reader) subjects

3. CATT whole blood positive (by at least one reader), HAT RDT negative subjects

4. CATT whole blood negative, HAT RDT negative subjects

For the purpose of allocating participants to these categories, an RDT or CATT test will be considered positive if at least one of the two independent readers reports a positive result. The results reported by each reader will be recorded separately for future retrospective analysis.

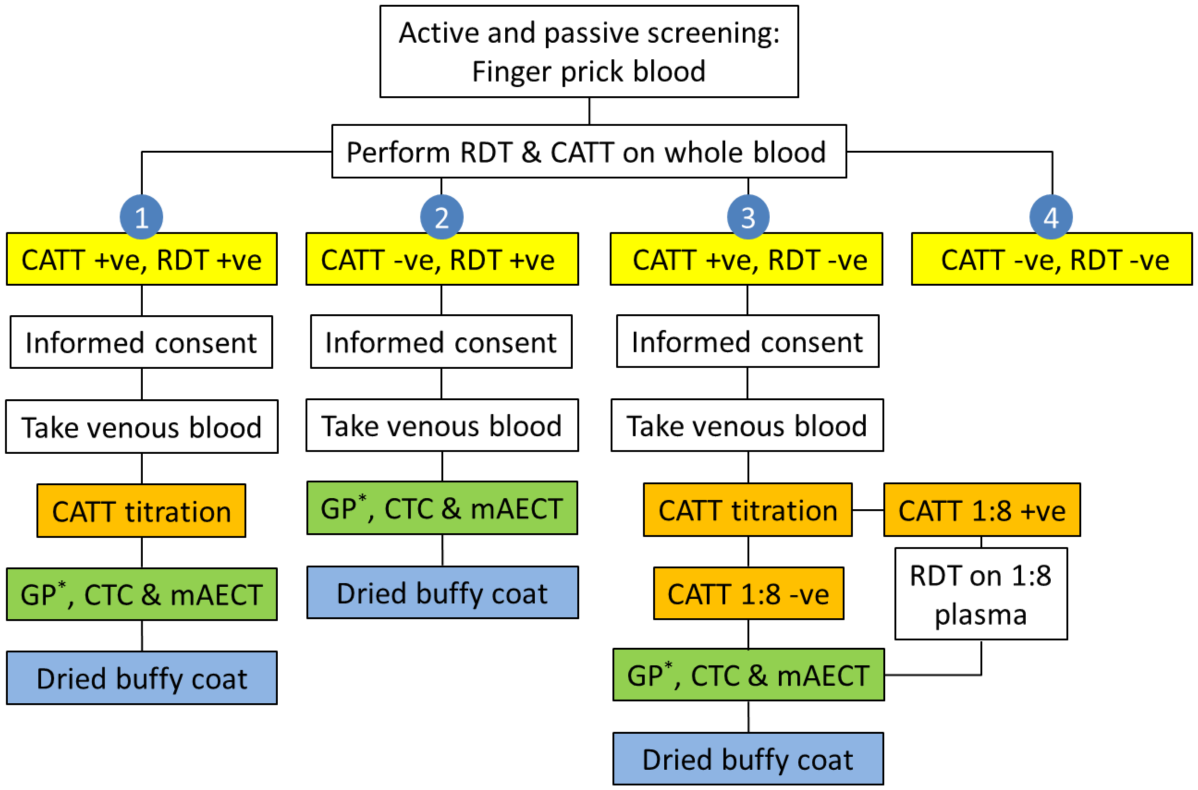

* GP (gland puncture) performed only if typical swollen cervical lymph nodes are found.

Figure 2. *Summary of procedures to be carried out in the study. Note that category 4 individuals presenting at health centres with clinical symptoms suggestive of HAT will be tested in the same manner as category 2 participants (see Point 9 above).*

11.2 Management of patients with positive test results

Only trypanosome positive individuals with parasitological confirmation by any of the routine methods will be considered as HAT cases. These will be treated according to the national control programme (PNLTHA) guidelines.

11.3 Follow-up

Individuals who are either CATT whole blood positive and/or RDT positive but who are negative by all parasitological methods and who will not be treated will be followed up. These individuals will be invited and/or actively looked for and will be tested again once after 3-6 months according to the same procedures as described under 11.1.

Since only a small number of individuals are expected to be followed up successfully during this study, the associated results will not be used to determine the accuracy of the diagnostic algorithms under investigation.

11.4 Cost analysis

The cost of diagnostic strategies including either the RDT and/or CATT will be evaluated either for passive or for active screening by determining the associated costs, including:

- Supplies (consumables, stationery)
- Equipment
- Shipment and storage

- Vehicles operation and maintenance (mobile teams)
- Personnel
- Training
- Laboratory space
- Transport/loss of income for individuals being screened.

11.5 Cost effectiveness

The cost effectiveness of diagnosing and treating HAT patients using various diagnostic algorithms including the RDT and/or the CATT test will be determined either for passive or for active screening. Methodologies similar to the ones described by Lutumba *et al.* (2005 and 2007) will be applied, which are described in detail in Appendix 1. Information on treatment costs will be collected from health centres based on past and/or on-going treatments.

11.6 Ease of implementation

The ease of implementation of the diagnostic strategies under investigation will be assessed by collecting and analysing the following information on the RDT and CATT:

**Information type**	**Information source**
Shipment needs	PNLTHA provincial coordinators (personal interviews)
Storage needs	PNLTHA provincial coordinators (personal interviews)
Personnel needs	PNLTHA provincial coordinators (personal interviews)
Need for specific equipment and accessories	PNLTHA provincial coordinators (personal interviews) & health workers (questionnaires)
Simplicity of performing test and reading results, including number of steps and timing	Health workers (questionnaires)
Safety (risk of contamination from blood)	Health workers (questionnaires)

12. Blinding

Blinding will be done at two levels. For initial screening of participants, one technician will be responsible for performing CATT tests, while in parallel, another technician will be testing participants with the RDTs. The two technicians will be working independently (but using blood from the same finger prick), without exchanging results (first level of blinding). A supervisor will be responsible for making sure that CATT and RDT testing will be done independently. Based on the CATT and RDT results, which will in turn be read by two independent readers, the supervisor will identify the participants from whom venous blood will be collected, according to the flowchart shown in Figure 2. Samples of venous blood will be labelled with blinding codes by the supervisor (second level of blinding). The same codes will be used to identify the buffy coat samples collected from the participants. All tests performed on these samples will be reported using blinding codes.

13. Ethical considerations

This study will be carried out in conformity with the Helsinki Declaration. The health centres and mobile teams have facilities and trained staff who will enrol participants and collect samples under GCLP conditions. The study will not involve a significant deviation from routine methods for sampling by the national HAT control programme; blood will be collected from a finger prick or by venipuncture, and no elaborate manipulations will be carried out on participants.

Written informed consent will be obtained from all participants from whom a venous blood sample will be drawn, i.e. for participants in categories 1, 2 and 3 (see Point 11.1 and Figure 2). An informed consent document has been prepared (see Appendix 2), which will be translated into local languages before initiation of the study.

14. Equipment and reagent needs

19,800 participants are planned to be tested with the RDT. Taking into account the needs for training and the possibility of getting invalid results, **20,000 SD BIOLINE HAT RDTs** will be supplied for this study. Similarly, **reagents to test 19,800 participants with CATT** will be needed (including sample dilutions).

RDT for malaria will be performed on all individuals who are positive by either CATT (whole blood) or by RDT, or both, as well as on all individuals presenting for passive screening. Thus around **4,500 malaria RDTs** will be necessary.

mAECT will be performed on all participants who are RDT and/or CATT whole blood positive, but who cannot be confirmed as HAT cases by gland puncture and/or CTC, corresponding to an expected total of approximately **2,000 mAECT tests**.

Equipment and supplies for other tests, such as microscopes and centrifuges, will be supplied by the PNLTHA as part of their routine activities.

15. Data management and analysis

Each enrolled subject will have a unique and anonymous identification (blinding code), initials, age, sex, and results of laboratory examination collected on a case report form (CRF) and laboratory results sheets. These documents will be filled in in single copies, which will remain at the study sites where they will be archived for at least 3 years after study completion. Data will be entered by data managers selected by the PI into a web-based database developed by FIND (VisionForm), which will be accessible for viewing by the PI, study monitor and FIND. Data entry will be performed at three sites: Dipumba for data from the East Kasai province, Bandundu for data from the Bandundu province, and X for data from the West Kasaï province. Endpoint values, as well as their corresponding 95% confidence intervals, will be calculated using SAS. For objective 1, the sensitivity of each diagnostic algorithm as well as the specificity of screening tests will be calculated as follows:

	Cases	Controls
Positive with diagnostic algorithm N	a	b
Negative with diagnostic algorithm N	c	d

	Cases	Controls

Positive with screening test X	e	f
Negative with screening test X	g	h

Sensitivity of HAT case detection using diagnostic algorithm N (i.e. 1, 2, 3 or 4) = a / (a + c)

Specificity of screening test X as part of diagnostic algorithm N = (b + d - f) / (b + d)

where the screening test X of diagnostic algorithm N is defined as follows:

	Diagnostic algorithm (N)			
	1	2	3	4
Screening test (X)	CATT whole blood	CATT 1:8	RDT	CATT 1:8

16. Minimization of bias

In order to minimize any bias, the study will be conducted according to the following principles:

- All samples will only be identified using blinding codes, which will ensure that technicians will have no information about the corresponding participants when analysing samples and recording results.
- Testing of samples with different methods (i.e. serology, parasitology) will be performed by independent technicians, who will not exchange or compare any results.

17. Quality assurance and quality control

Diagnostic tests, data entry and management will be conducted according to SOPs and as described in the relevant sections of the protocol. Expiry dates of reagents and test kits will be checked prior to use. All buffy coat samples collected on filter papers will be analysed within 4 weeks of sample collection. Shipments of RDTs will be accompanied by a LogTag, which will measure and record ambient temperature every hour. One LogTag will also be kept with the stock of RDTs at each health centre and by each mobile team during the course of the study.

18. Trial site assessment

All study sites will be inspected by a FIND employee or consultant prior to initiation of the study, according to the procedures described in the FIND Clinical Trial Standard. Laboratory and clinical quality standards of sites will be assessed using standardized questionnaires.

19. Study coordination, training and monitoring

FIND is the study sponsor and is responsible for planning and managing the study. Dr. Joseph Ndung'u will be the FIND Study Coordinator. Dr. Crispin Lumbala (PNLTHA, DRC) will be the Principal Investigator (PI).

The sites study team will receive on-site technical training by the PI and FIND during study initiation visits. The training will also include completion of CRFs, laboratory results sheets,

questionnaires, and data management. Study staff will be trained in checking study inclusion criteria, obtaining informed consent, and will be familiarized with the study protocol and related SOPs. A brief pilot period will be included before beginning the study, which will allow the study personnel to identify and resolve potential technical or operational problems prior to beginning data collection.

The daily activities related to the study implementation in the different provinces will be directly supervised and coordinated by the PI.

An independent study monitor will be hired by FIND to visit the study sites at study initiation, after 3 weeks of enrolment, after 7 weeks of enrolment and upon completion of the study. During his visits, the monitor will control adherence to the study protocol and SOPs, and will verify the completed CRFs and laboratory results sheets against the source documents.

The FIND Study Coordinator will be in constant contact with the study staff to assess progress of the study, to ensure consistency in the collection of data, and to address any difficulties with the laboratory procedures or protocol.

20. Dissemination of results and publication policy

The findings of this project will be published in peer-reviewed journals. The Principal Investigator and FIND will compile and analyse the data from all the sites and prepare a first draft for review by the study sites and partners. The results of the analysis will be subjected to a further review by an external expert to be contracted by FIND. All scientific manuscripts, reports, presentations or communications related to this project shall be sent to FIND for review and approval prior to any publication.

21. Risks and mitigation steps

Given the rapidly decreasing prevalence of HAT in the study areas, enrolling 242 cases within 8 weeks might represent a challenge. To mitigate this risk, the number of enrolled cases will be monitored fortnightly, and if a significant deviation from targets is identified, active screening activities will be intensified as necessary, to cover a larger proportion of the population at risk.

References

Blackwelder WC (1982). Proving the null hypothesis in clinical trials. Controlled Clinical Trials 3:345-353.

Checchi F, Chappuis F, Karunakara U, Priotto G, Chandramohan D (2011). Accuracy of five algorithms to diagnose gambiense human African trypanosomiasis. PLoS Negl Trop Dis. 5(7):e1233.

Chappuis F, Lima MA, Flevaud L, Ritmeijer K (2010). Human African Trypanosomiasis in Areas without Surveillance. Emerging infectious diseases 16(2):354-355.

Deborggraeve S, Büscher P (2010). Molecular diagnostics for sleeping sickness: what is the benefit for the patient? Lancet Infect Dis 10(6):433-9.

Kennedy PG (2008). Diagnosing central nervous system trypanosomiasis: two stage or not to stage? Trans R Soc Trop Med Hyg 102(4):306-7.

Lutumba P, Robays J, Miaka C, Kande V, Simarro PP, Shaw AP, Dujardin B, Boelaert M (2005). Efficience de différentes stratégies de détection de la Trypanosomiase Humaine Africaine à *T. b. gambiense*. Trop Med Int Health 10(4):347-56.

Lutumba P, Meheus F, Robays J, Miaka C, Kande V, Büscher P, Dujardin B, Boelaert M (2007). Cost-effectiveness of algorithms for confirmation test of human African trypanosomiasis. Emerg Infect Dis 13(10):1484-90.

Simarro PP, Diarra A, Ruiz Postigo JA, Franco JR, Jannin JG (2011). The human African trypanosomiasis control and surveillance programme of the World Health Organization 2000–2009: The way forward. PLoS Negl Trop Dis 5(2):e1007.

Steverding D (2006). A new initiative for the development of new diagnostic tests for human African trypanosomiasis. Kinetoplastid Biol Dis 5:1.

APPROVAL

______________________________	____________________
Study Coordinator	Date
______________________________	____________________
HPED	Date
